# Tachyarrhythmias, bradyarrhythmias and acute coronary syndromes

**DOI:** 10.4103/0974-2700.62112

**Published:** 2010

**Authors:** Hans-Joachim Trappe

**Affiliations:** Department of Cardiology and Angiology, University of Bochum, Germany

**Keywords:** Acute coronary syndrome, bradyarrhythmias, defibrillation, electrostimulation, tachyarrhythmias

## Abstract

The incidence of bradyarrhythmias in patients with acute coronary syndrome (ACS) is 0.3% to 18%. It is caused by sinus node dysfunction (SND), high-degree atrioventricular (AV) block, or bundle branch blocks. SND presents as sinus bradycardia or sinus arrest. First-degree AV block occurs in 4% to 13% of patients with ACS and is caused by rhythm disturbances in the atrium, AV node, bundle of His, or the Tawara system. First- or second-degree AV block is seen very frequently within 24 h of the beginning of ACS; these arrhythmias are frequently transient and usually disappear after 72 h. Third-degree AV blocks are also frequently transient in patients with infero-posterior myocardial infarction (MI) and permanent in anterior MI patients. Left anterior fascicular block occurs in 5% of ACS; left posterior fascicular block is observed less frequently (incidence <0.5%). Complete bundle branch block is present in 10% to 15% of ACS patients; right bundle branch block is more common (2/3) than left bundle branch block (1/3). In patients with bradyarrhythmia, intravenous (IV) atropine (1-3 mg) is helpful in 70% to 80% of ACS patients and will lead to an increased heart rate. The need for pacemaker stimulation (PS) is different in patients with inferior MI (IMI) and anterior MI (AMI). Whereas bradyarrhythmias are frequently transient in patients with IMI and therefore do not need permanent PS, there is usually a need for permanent PS in patients with AMI. In these patients bradyarrhythmias are mainly caused by septal necrosis. In patients with ACS and ventricular arrhythmias (VTA) amiodarone is the drug of choice; this drug is highly effective even in patients with defibrillation-resistant out-of-hospital cardiac arrest. There is general agreement that defibrillation and advanced life support is essential and is the treatment of choice for patients with ventricular flutter/fibrillation. If defibrillation is not available in patients with cardiac arrest due to VTA, cardiopulmonary resuscitation is mandatory.

## INTRODUCTION

Treatment of patients with acute coronary syndrome (ACS) is sometimes difficult and can pose major problems for the treating physician.[1–4] The earliest change in the electrocardiogram (ECG) in acute cardiac ischemia following a coronary occlusion is deviation of the ST segment from the isoelectric line. The location and severity of ischemia are indicated by the amount and direction of the ST segment deviation.[5] Failure to restore blood flow at this time will be followed by loss (necrosis) of myocardial tissue, with changes in the QRS and the T wave. In addition to ST segment changes, bradyarrhythmias (heart rate < 50 bpm) or tachyarrhyhmias (heart rate > 100 bpm) are frequently present in acute myocardial ischemia and this can lead to further ischemia, with or without left ventricular dysfunction.[6–8] In acute myocardial infarction (MI) sudden cardiac death due to ventricular tachycardia, ventricular flutter, or ventricular fibrillation occurs in approximately 50% of all patients as the first sign of coronary artery disease.[9,10] In Germany, approximately 100000 patients die suddenly per year; this sudden death is caused by ventricular tachyarrhythmias in 65% to 80% of these patients, whereas bradyarrhythmias are present in 5% to 20% of them. To avoid myocardial necrosis or sudden cardiac death it is necessary to recognize the high-risk patient. Typical ECG changes can indicate the severity of cardiac ischemia and the site of occlusion of coronary arteries.[11,12] Electrical treatment of brady- or tachyarrhythmias is often necessary to treat patients with acute MI and to avoid ‘electrical problems,’ left ventricular impairment, or sudden cardiac death.

## BRADYARRHYTHMIAS IN ACS

Bradyarrhythmias are often dangerous in patients with ACS in whom sufficient myocardial function is no more possible.[13,14] The incidence of bradyarrhythmias like sinus node dysfunction (SND), sinoatrial conduction abnormalities, higher degree AV blocks, or bundle branch blocks ranges between 0.3% and 18%.[15] The emergence of conduction disturbances between the sinus node and the right atrium and between the atrium and ventricles in the acute phase of MI is of prognostic and therapeutic significance. The site of block is related to the particular occluded coronary artery.

## BLOOD SUPPLY TO THE CONDUCTION SYSTEM

The sinus node and the sinoatrial region are, in 55% of patients, perfused by an atrial branch from the proximal part of the right coronary artery (RCA). In 45% of cases this region is perfused by a proximal branch of the circumflex coronary artery.[16] A proximal occlusion of the RCA or the cicumflex coronary artery may therefore lead to ischemia of the sinus node and the surrounding atrium. The AV conduction system (the AV node, the bundle of His, and the bundle branches) is perfused by the RCA and the left anterior descending coronary artery (LAD); the AV node and the proximal part of the bundle of His are perfused by the RCA, while the distal part of the bundle of His, the right bundle branch, and the anterior fascicle of the left bundle branch are supplied by the septal branches of the LAD. The posterior fascicle of the left bundle branch is supplied by septal branches from both the LAD and the RCA.[16]

## SINUS NODE DYSFUNCTION

**Figure 1:**
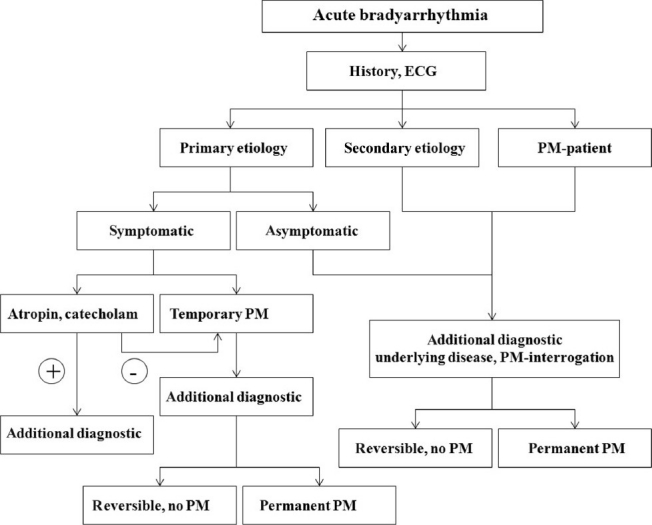
Treatment algorithm in patients with bradyarrhythmia. Abbreviations: Perman = permanent, PM = pacemaker

**Figure 2:**
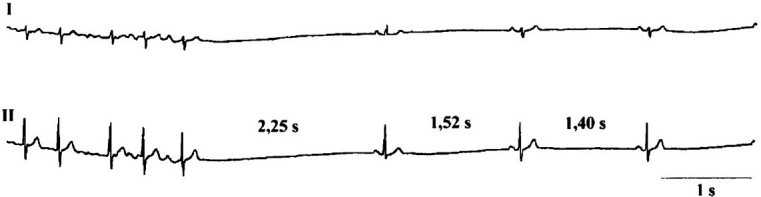
Monitor-ECG in a patient with acute coronary syndrome and bradyarrhythmias due to sinus node arrest

There are several conduction disturbances that arise from the sinus node or the surrounding area. These arrhythmias are sinus bradycardia, sinoatrial block, sinus arrest, or bradycardia/ tachycardia syndromes [Figures 1 and 2]. Possible mechanisms for sinus bradycardia after an acute MI are neurologic reflexes (Bezold-Jarisch reflex), coronary chemoreflexes (vagally mediated), humoral reflexes (enzymes, adenosine, potassium), oxygen conserving reflex (‘diving’ reflex), or infarction or ischemia of the sinus node or the surrounding atrium. SND with or without sinus bradycardia is frequently observed in patients with infero-posterior MI and, in general, such sinus bradycardia indicates a smaller infarct because it is usually vagally induced.

### Acute management

**Figure 3:**
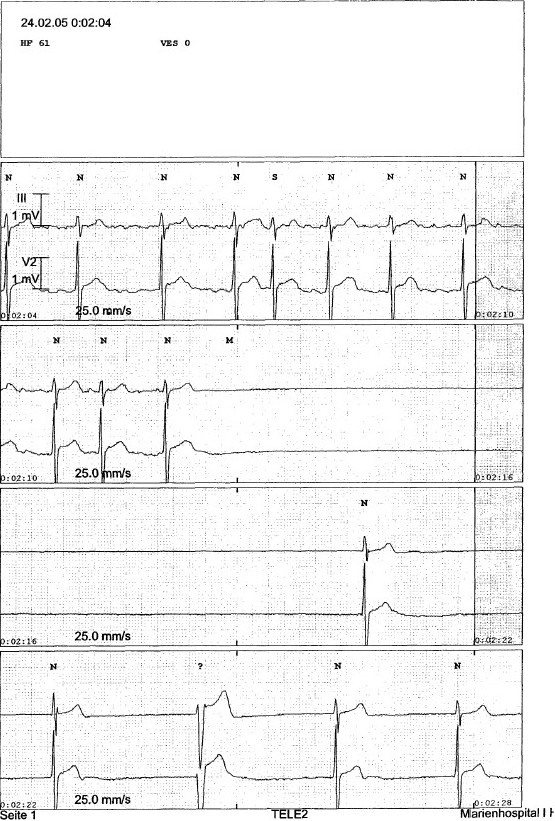
Brady-tachy syndrome in a patient with acute inferior myocardial infarction and asystole until 2.25 s

In patients with SND, temporary pacing is indicated if, despite vagolytic therapy, hypotension, dizziness, or presyncope are present [Figure 3]. In patients with sinoatrial block or sinus arrest, pacing is indicated when the slow heart rate leads to low cardiac output or increased ventricular ectopic impulse formation.

## AV NODAL CONDUCTION DISTURBANCES

In ACS, conduction abnormalities occur more frequently than SND or sinus node conduction disturbances.[17] Conduction disturbances at the level of the AV node are usually seen in cases of occlusion of the RCA proximal to the right ventricular branch. First-degree AV blocks are observed in 4% to 13% of patients with acute MI and occur due to conduction delays in the atrium, AV node, bundle of His, or the Tawara system, particularly in patients with infero-posterior ischemia. The occurrence of high-degree (second-degree or more) AV nodal block in the setting of acute MI has important prognostic implications, with the mortality rate being 2–3 times greater when high-degree AV block is present [Table 1]. High-degree AV block can be caused by increased vagal tone or it may be the result of myocardial necrosis in the AV node or the surrounding tissue. High-degree AV blocks can follow first-degree AV block and may be transient. Third degree AV block is present in 3% to 19% of patients with acute MI. AV nodal block (first- or second-degree AV nodal block) are frequently present within 24 h after the start of myocardial ischemia and is usually transient; AV nodal block after inferior MI is rarely permanent. The occurrence of complete AV nodal block after inferior MI is also frequently transient and in most of these patients there is normal AV conduction 3–7 days later.

**Table 1:** AV conduction disturbances in acute myocardial infarction (second-degree or worse)

	Anterior	Inferoposterior
Culprit coronary artery	LAD	RCA
Escape rhythm	Wide QRS	Narrow or wide QRS
	HR < 40 beats/min	HR 40-60 beats/min
Duration	Usually transient	Usually transient
Incidence	5%	12-20%
Mortality (compared with mortality in those with no conduction disturbance)	2-3 times	4 times

HR = HEART RATE, LAD = LEFT ANTERIOR DESCENDING CORONARY ARTERY, MIN = MINUTE, RCA = RIGHT CORONARY ARTERY

### Acute management

Conduction abnormalities at the AV nodal level with second-degree or worse AV conduction disturbances in acute MI stresses the importance of early reperfusion, preferably via percutaneous coronary intervention. AV nodal block after inferior MI is rarely permanent and pacing is only indicated when symptomatic second-degree or complete AV nodal block persist more than 2 weeks after inferior MI. The decision to pace will depend on the clinical signs and electrocardiographic findings [Table 2].

**Table 2:** Indications for temporary or permanent pacing in patients with acute myocardial infarction

	Temporary pacing	Permanent pacing
SN dysfunction	Symptomatic pt No response to atropine	Symptomatic pt with sinuatrial block
AV block	AV-block III° without sufficient escape rhythm	Persistent AV block III° in IMI (> 10 days postinfarct)
	- Symptomatic pt	
	- Ventricular irritability	Persistent AV block III° in AMI
	- Hemodynamic deterioration	
	Symptomatic pt with AV block II°	Symptomatic pt with persistent AV block II°
BBB	AMI with new LBBB,	Alternating LBBB/RBBB
	Hemodynamic instability	
	Alternating LBBB/RBBB	
	RBBB + LAFB/LPFB	Symptomatic pt with
	LBBB + AV block I°	RBBB + LAFB/LPFB

AMI = ANTERIOR MYOCARDIAL INFARCTION, IMI = INFERIOR MYOCARDIAL INFARCTION, LAFB = LEFT ANTERIOR FASCICULAR BLOCK, LPFB = LEFT ANTERIOR FASCICULAR BLOCK, LBBB = LEFT BUNDLE BRANCH BLOCK, PT = PATIENT, RBBB = RIGHT BUNDLE BRANCH BLOCK

## CONDUCTION DISTURBANCES BELOW THE AV NODE

The development of conduction disturbances in or below the bundle of His in association with acute MI is a specific marker for a very proximal occlusion of the LAD and therefore indicates that a large area of the left ventricle is in jeopardy. There is a poor prognosis for patients with an acute MI and conduction disturbances below the AV node [Table 1]. Left anterior fascicular block occurs in approximately 5% of patients with acute myocardial ischemia; left posterior fascicular block is less frequently observed (incidence < 0.5%).[18] The most common type of sub-AV nodal conduction disturbance following an LAD occlusion proximal to the first septal branch is right bundle branch block (RBBB) (QRS width ≥0.12 s) with or without left fascicular block. This disturbance is much more common than the development of complete left bundle branch block (LBBB). When RBBB is the result of the MI, the prognosis is ominous, especially when accompanied by left fascicular block.[19–22] When a patient presenting with acute MI also has RBBB, discerning whether the block was already present before the MI or was caused by the infarction is important with regard to the prognosis. When the RBBB was preexisting, hospital mortality rate was no different from that of patients without RBBB. In contrast, RBBB that results from an MI (typically, a very proximal LAD occlusion) indicates a poor prognosis.[23]

### Acute management

In patients with conduction disturbances below the AV node, reperfusion therapy (via percutaneous coronary intervention) should be performed in the patient with LBBB when the clinical presentation suggests acute MI. Whether temporary or permanent pacing is to be done depends on the clinical signs and the electrocardiographic findings [Table 2].

## VENTRICULAR TACHYARRHYTHMIAS IN ACS

Among the most important problems in intensive care and in emergencies is the patient with recurrent ventricular tachycardia, ventricular flutter, or ventricular fibrillation.[24] Management of cardiac arrest due to life-threatening ventricular tachyarrhythmias is a very important goal in these patients in order to avoid serious problems and to avoid sudden cardiac death.[25] However, treatment of the underlying arrhythmia requires correct diagnosis which, in the majority of patients, is possible with the aid of a 12-lead surface ECG.[26] However, careful interpretation of all 12 ECG leads is necessary for correct diagnosis, both in bradyarrhythmia and tachyarrhythmia. Because a drug given for the treatment of supraventricular tachycardia may be deleterious to a patient with ventricular tachycardia, the differential diagnosis in broad-QRS tachycardia is critical. To make the right diagnosis, it is ideal to have a 12-lead ECG. Diagnostic clues for differentiation of VT from SVT are found in leads V1 and V6; in addition, a QRS of 0.14 s or more favors a diagnosis of VT. There are several possible mechanisms of wide-QRS-complex tachycardia. In intensive care and emergencies it is necessary to divide wide-QRS-complex tachycardia into those with monomorphic morphology and those with polymorphic morphology; in addition there are those with torsade de pointes tachycardia.

### Acute management

The initial approach depends on the hemodynamic severity and the symptoms associated with the tachycardia. When the patient is hemodynamically unstable or has pulmonary edema, the tachycardia should be promptly cardioverted with a direct current synchronized shock. Once the hemodynamically stable patient has been cardioverted and stabilized, it is important to evaluate the preconversion 12-lead ECG for QRS configuration and signs of AV dissociation. For hemodynamically stable patients, a 12-lead ECG should allow an accurate diagnosis in the majority of patients. If after analyzing the ECG the diagnosis remains uncertain, the patient should be treated for VT. This is by far the most common diagnosis in patients with wide-QRS-complex tachycardia and VT is potentially life-threatening. In patients with sustained (duration > 30 s) hemodynamically stable monomorphic VT, amiodarone (150-300 mg in 5 min IV, followed by an infusion of 1050 mg/day) plays an important role in terminating the attack. Alternatives are procainamide (10 mg/kg IV) or ajmaline (50-100 mg IV over 5 min), both of which are associated with high termination rates. In patients with VT and in the setting of acute myocardial ischemia, lidocaine (100-150 mg IV) was the treatment of choice for a long time. However, it is well known that ajmaline is more effective than lidocaine. Moreover, lidocaine is associated with high risk for degeneration of monomorphic VT into ventricular fibrillation. Therefore, lidocaine is no longer indicated in these patients and should be avoided. Other antiarrhythmic drugs like sotalol (20 mg in 5 min IV), propafenone (1-2 mg/kg IV) or flecainide (1-2 mg/kg IV) do not have any role as ‘first-line’ drugs in stable monomorphic VT.[27–29] The treatment of choice for polymorphic VT is isoproterenol infusion, which shortens the repolarization period and increases the heart rate (1-4 µg/min IV) as initial steps. An alternative treatment is atropine (0.5-1.0 mg IV, up to a maximum of 0.04 mg/kg IV). Atrial or ventricular pacing will often suppress polymorphic VT. In these situations, the presence of polymorphic VT is a clear indication for emergency treatment with amiodarone (150-300 mg IV, followed by an infusion of 1020 mg/day).

## TORSADE DE POINTES TACHYCARDIA

Torsade de pointes is a type of polymorphic VT; it is associated with marked QT prolongation. It may occur after administration of class Ia and class III antiarrhythmic drugs. The tachycardia is paroxysmal and may result in ventricular fibrillation and sudden death.[30,31] Its onset is promoted by a slow basic rhythm and frequently follows a pause induced by a premature ventricular contraction. The tachycardia is characterized by polymorphic QRS complexes. Progressive lengthening of the QT interval and the development of a prominent U wave are important warning signs. The degree of QT prolongation that predicts torsade de pointes tachycardia is not known. However, prolonged QT syndromes may be congenital (Romano-Ward syndrome or Jervell-Lange-Nielsen syndrome) or acquired (class I and class III antiarrhythmic drugs). Intravenous magnesium sulfate (an initial bolus of 2 g IV, with another bolus of 2 g after 15 min if the initial bolus fails, followed by a continuous infusion of 500 mg/h IV) may be efficacious even if the serum magnesium level is within the normal range.[32,33] Although magnesium has been used to treat arrhythmias for several decades, its mechanism of action and efficacy remain controversial.[34,35] The use of magnesium for this purpose has not undergone scrutiny in randomized trials.[36]

## VENTRICULAR FLUTTER AND VENTRICULAR FIBRILLATION

Approximately 1000 people in the United States suffer from cardiac arrest each day, most often as a complication of an acute MI with accompanying ventricular fibrillation or unstable VT. In 2005, the American Heart Association (AHA) reported again the ‘chain of survival’ concept, with four links – early access, cardiopulmonary resuscitation, defibrillation, and advanced care – as the way to approach cardiac arrest.[37] It has been pointed out that the highest potential survival rate from cardiac arrest can be achieved only when the following sequence of events occurs as rapidly as possible: (a) recognition of early warning signs, (b) activation of the emergency medical services system, (c) basic cardiopulmonary resuscitation, (d) defibrillation, (e) management of the airway and ventilation, and (f) intravenous administration of medications. Of course, defibrillation is the treatment of choice in patients with ventricular flutter or ventricular fibrillation. It is one of the most promising interventions for achieving improved survival from cardiac arrest.

## CONCLUSIONS AND CLINICAL IMPLICATIONS

The ECG is of great value in patients with acute MI and is essential for risk stratification and selection of optimal management. For risk stratification, different ECG parameters are helpful; for example, ST segment deviation score, severity of ischemia as indicated by the ST-T segment behavior, ST segment deviation vector in the frontal plane to detect the site of occlusion in the culprit coronary artery and estimate the size of the area at risk, and the presence of sinoatrial, AV nodal, or intraventricular conduction disturbances. There are several typical findings of brady- or tachyarrhythmias that are associated with an increased risk for heart failure and/or sudden cardiac death. Careful analysis of the 12-lead ECG, with right decision-making in emergencies, will lead to better patient survival.
